# High Host Preferences in Epiphytic Lichens Across Diverse Phorophyte Species in the Mediterranean Region

**DOI:** 10.3390/jof11020104

**Published:** 2025-01-30

**Authors:** Gregorio Aragón, Isabel Martínez, Marcelino de la Cruz, Pilar Hurtado

**Affiliations:** 1 Biodiversity and Conservation Area, Department of Biology, Geology, Physics and Inorganic Chemistry, ESCET, Rey Juan Carlos University, c/Tulipán s/n, Móstoles, 28933 Madrid, Spain; isabel.martinez@urjc.es (I.M.); marcelino.delacruz@urjc.es (M.d.l.C.); pilar.hurtado@urjc.es (P.H.); 2 Global Change Research Institute, Rey Juan Carlos University, Móstoles, 28933 Madrid, Spain

**Keywords:** epiphytes, lichens, host preferences, *Quercus*, *Juniperus*, Iberian Peninsula

## Abstract

Contrary to the assumption that epiphytic lichens, which obtain water and nutrients from the atmosphere, do not exhibit host species preference, this notion is challenged by the limited number of studies that cover a wide geographical range and diverse phorophyte species (hereafter referred to as “host species”). To investigate this assumption, we evaluated the host preference of 709 epiphytic lichen species across the Mediterranean basin, examining 72 host species. The research is based on field studies conducted by the authors, supplemented with bibliographic records to expand the study area and the number of host species. We define “host preference” as the association of an epiphytic lichen species with a single host species. Our findings reveal a high prevalence of lichens exhibiting host preference both locally (exceeding 30% of lichen species in each of six geographic areas) and regionally (25% across the entire dataset). This host preference remained consistent even with increased sampling extent, which can be attributed to factors such as hosts with diverse bark types, the wide climatic range of some species, and host species associated with extreme environmental conditions within the Mediterranean region. Overall, we conclude that host bias for epiphytic lichen species remains consistent in Mediterranean landscapes, contributing to a diverse array of epiphytic species and high levels of host species preference. This research provides valuable insights into the complex interactions between lichens and their host species, offering a deeper understanding of biodiversity within Mediterranean landscapes.

## 1. Introduction

The role of phorophyte species (hereafter referred to as “host species”) in shaping the composition of epiphytic lichen communities has been demonstrated in several studies conducted under varying environmental conditions [1,2,3,4]. Despite their symbiotic nature and dependence on water and nutrients primarily obtained from the atmosphere, lichens are not independent of their substrate. The diverse bark conditions of host species provide varied habitats for epiphytic lichens [5,6]. This relationship is closely tied to host traits, including chemical and physical bark characteristics (such as pH, texture, roughness, water-holding capacity, and nutrient content), as well as canopy structure and foliage density [1,4,5,7,8,9]. Consequently, lichen species exhibit preferences for specific host traits, leading to the expectation that forests with different tree species compositions may harbour distinct lichen species [10,11].

The distribution of epiphytic lichens among host species results from the tendency of an epiphytic species to be more abundant on specific host species. Thus, while most epiphytic lichens exhibit a generalist strategy, growing on a wide variety of host species (e.g., *Parmelia sulcata*, which thrives equally on coniferous and deciduous tree species), some lichens exhibit preferences based on host similarity (e.g., certain epiphytic species such as *Platismatia glauca* predominantly grow on different coniferous species) [7].

However, the influence of host tree identity on epiphytic lichens remains an open and controversial issue [11]. Over the years, several authors have suggested that the relatively low number of lichen species shared among host tree species, compared to those exclusively found on each host, could be attributed to the preferences of epiphytic lichens for particular host species [4,11,12,13,14,15]. These lichen–host preferences can be quite common and can account for over 50% of lichen species in some cases. These outcomes that overvalue the host preference rates could be related to (a) studies conducted over limited geographical areas [4,14,15], (b) studies involving only a small number of host species [11,15,16,17,18,19], or (c) studies considering tree species with different bark characteristics, such as plantations versus native forests [13,20] or *Quercus* versus *Pinus* species.

In this context, the primary objective of this study is to determine the true prevalence of host preference among lichen epiphytes across a broad geographical area and a substantial number of phorophyte species. Given that this research was conducted within a regional context partially covering the Mediterranean region of Spain, we prefer to use the term “host preference” rather than “host specificity” when an epiphytic lichen species is identified on a single host species. To address this, we investigated various forests and scrublands within the Mediterranean basin, an area characterized by its heterogeneous landscape, diverse land uses (such as timber and firewood extraction, deforestation for agricultural expansion, extensive cattle grazing, and hunting management), and a high degree of variation in forest architecture, growth forms, leaf types, and woody plant species [21,22,23]. Considering these factors, our hypothesis suggests that given the geographical scope (climatic range) of the study and the high diversity of phorophytes (different types of tree bark), several lichen species will exhibit a preference for specific host species. Furthermore, we expected that host preference would be stronger in phorophytes with a wide geographic range (*Quercus ilex*), bark types that are very different (*Quercus suber*) compared to other phorophytes, or those phorophytes associated with extreme environmental conditions within the Mediterranean region (*Juniperus thurifera*).

To provide a comprehensive understanding of the Mediterranean landscape, it is essential to consider the predominant vegetation types and their influence on epiphytic biota. Mediterranean vegetation is predominantly composed of sclerophyllous forests (*Quercus ilex*, *Q. suber*), either monospecific or mixed, often accompanied by xerophytic conifers. In more humid areas, marcescent forests (*Quercus faginea*, *Q. pyrenaica*) become more prominent, especially at higher altitudes or in more northern regions. Mediterranean coniferous forests are also common both at lower elevations and in mountainous areas, with species such as *Pinus halepensis*, *P. nigra*, *P. pinaster*, *P. pinea*, *P. sylvestris*, and *Juniperus thurifera*. In their natural habitats, these forests are characterized by low density and coexistence with shrub vegetation [24]. The composition of epiphytic biota is highly determined by the forest type [25]. In low-altitude, warmer sclerophyllous forests that experience some degree of exploitation, parmelioid species such as *Melanelixia*, *Melanohalea*, and *Parmelina*, as well as *Physcia* and *Physconia*, are predominant [26,27]. In more humid environments, the bark of marcescent tree species is predominantly covered by parmelioid species such as *Usnea*, *Pertusaria*, and *Ochrolechia*, along with cyanolichens such as *Collema*, *Leptogium*, and *Lobarina* [27,28]. Montane coniferous forests are predominantly covered by *Usnea*, *Parmelia*, *Pseudevernia*, *Plastismatia*, and *Hypocenomyce* species. In more humid and well-preserved areas, these forests are further enriched with pin lichens such as *Calicium* and *Chaenotheca* species [29].

## 2. Materials and Methods

### 2.1. Data Set

This research is primarily based on numerous records derived from studies on epiphytic lichens conducted by the authors over the past 25 years (1998–2023) in the Mediterranean region of the Iberian Peninsula. Additionally, bibliographic records from other authors were included to expand the studied area and the number of host species. For this purpose, a bibliographic review was carried out using the ‘Checklist of the Iberian Lichens’ [30] and its updates [31,32].

A total of 90 papers were retained following a meticulous screening of titles, abstracts, and species lists based on the following criteria: (i) papers focused on epiphytic lichens, (ii) conducted in the Iberian Mediterranean region, (iii) providing clear and precise geographic location information, and (iv) offering clear information about the association between lichens and host species (tree or shrub).

**Figure 1:**
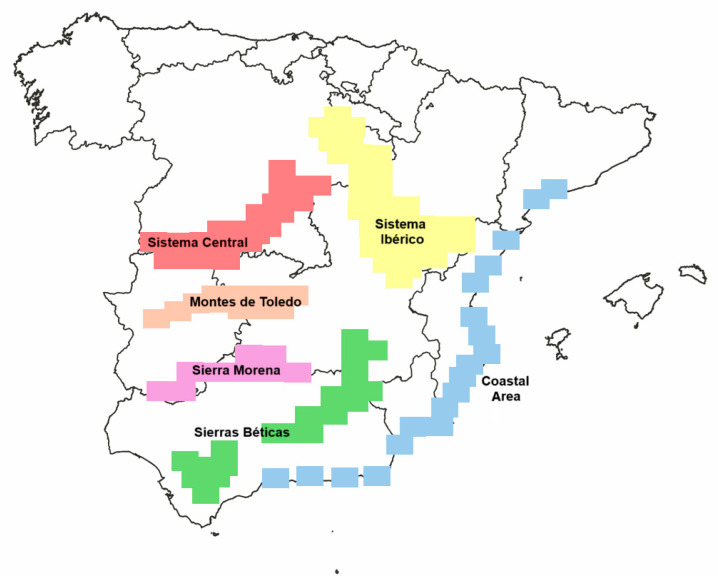
Map showing the location of the six geographics zones in Spain: Montes de Toledo, Sierra Morena, Sistema Central, Sierras Béticas, Sistema Ibérico, and Costal Area.

The final dataset comprises 35,366 records, including 709 lichen species on 72 host species. Each record includes information on the presence/absence of lichen species, host species, and geographic data, such as the province and geographic areas representing the five main mountain systems, along with the coastal area of the Iberian Mediterranean Region (Figure 1). Additionally, localization is provided using 10 km x 10 km UTM. Each UTM can include one or more host species, and the same host species can appear in multiple UTMs. We have considered several species that typically inhabit soil and rocks but are also found on bark.

### 2.2. Data Analyses

The dataset was examined at two distinct levels: host species and geographic area. At the host species level, we determined the prevalence of host preference among epiphytic lichens by calculating the number of lichen species exclusive to a single host species at the regional level (i.e., considering the entire dataset). Similarly, at the local level (geographic area), we calculated the prevalence of host preference by determining the number of lichen species exclusive to a single host species in each of the six geographic areas.

For each geographic area, we also generated UTM-based “host preference” accumulation curves [33], which illustrated the increase in the number of lichen species growing on a single host species as sampling effort increased in each area (i.e., as more 10 × 10 km UTM cells are considered). At the onset of sampling, numerous exclusive species were obtained since the epiphytic species growing on very few host species were compared, thereby increasing the slope of the curve. We anticipated that as more UTM cells were incorporated, the curve would stabilize at an asymptote. Therefore, we utilized exclusivity at the UTM level by randomly adding the UTMs and plotting the curves as the average host preference in 99 random subsets for each number of UTM cells. Accumulation curves were generated for each geographic area (local level) and for the entire region (regional level using the total dataset). Using these curves, we could assess the variation of lichen preference in each geographic area (i.e., how variable the exclusivity is as the number of UTMs increases). Calculations were performed using the vegan package [34] in R [35].

Finally, we selected *Quercus ilex* as a model species to demonstrate, using a Venn diagram, the exclusive versus shared species for *Q. ilex* among the six geographic areas considered. We chose *Q. ilex* because it is the host species with the largest distribution area, it appears in a greater number of UTMs, and it is present in all six regions considered.

In Table 1, the number of UTMs, phorophyte species, and lichen species across the six geographic areas considered are included. Table 2 lists the phorophyte species with at least five exclusive lichen species. This threshold was chosen to ensure clarity and simplicity in data presentation, as well as to highlight the ecological relevance of the phorophyte species.

## 3. Results

A total of 709 epiphytic lichen species were recorded on 72 host species across a large area of the Mediterranean region, covering 486 UTMs at a 10 km × 10 km resolution. The most frequently encountered host species were *Quercus ilex*, *Q. faginea*, and *Q. pyrenaica*, which occurred in 202, 109, and 109 UTMs, respectively (as detailed in Table 2 and Table S1). Among the epiphytic lichens, those with the widest host range included *Xanthoria parietina*, *Lecanora chlarotera*, and *Lecidella elaeochroma*, each found on 42, 44, and 52 host species, respectively (Table S1). Moreover, the percentage of lichen species restricted to a single host species was remarkably high, exceeding 30% in all six geographical areas considered (as summarized in Table 1). When analysing the entire dataset spanning 486 UTMs, this percentage remained at 25%.

**Figure 2:**
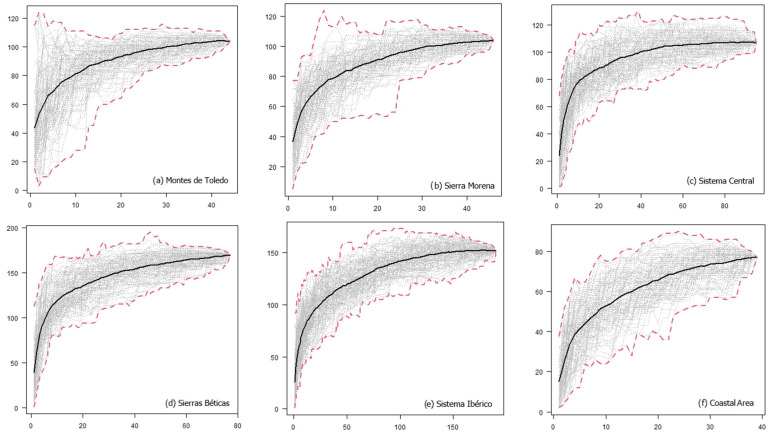

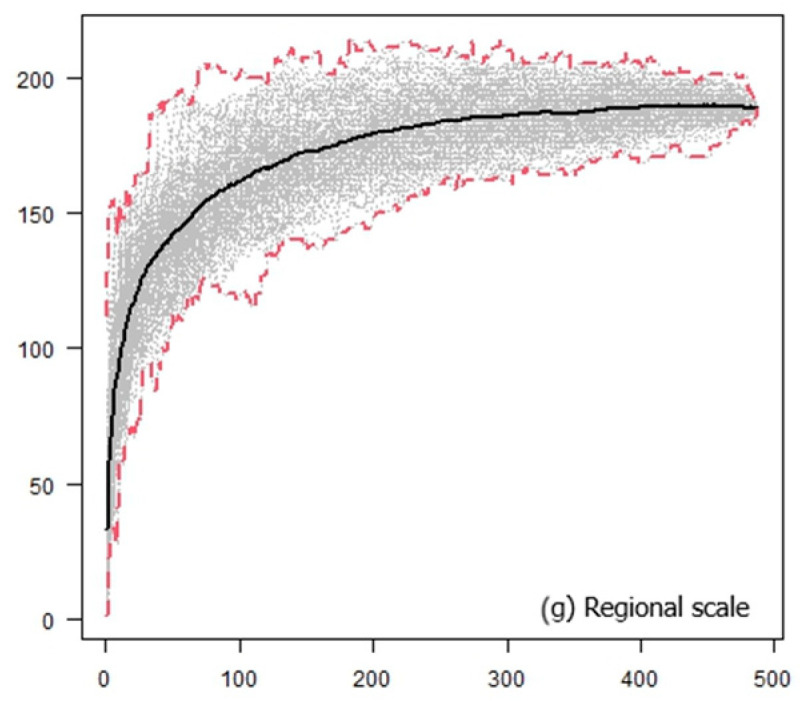
Host preference accumulation curves at the local (**a**)–(**f**) and regional (**g**) levels. Black lines represent the average of 99 permutations of the order of incorporation of the UTMs, while dashed red lines depict the maximum and minimum values observed in the set of permutations. The abscises axis represents the number of UTM 10 × 10 km, and the ordinate axis represents the number of lichen species growing on a single host species.

**Figure 3:**
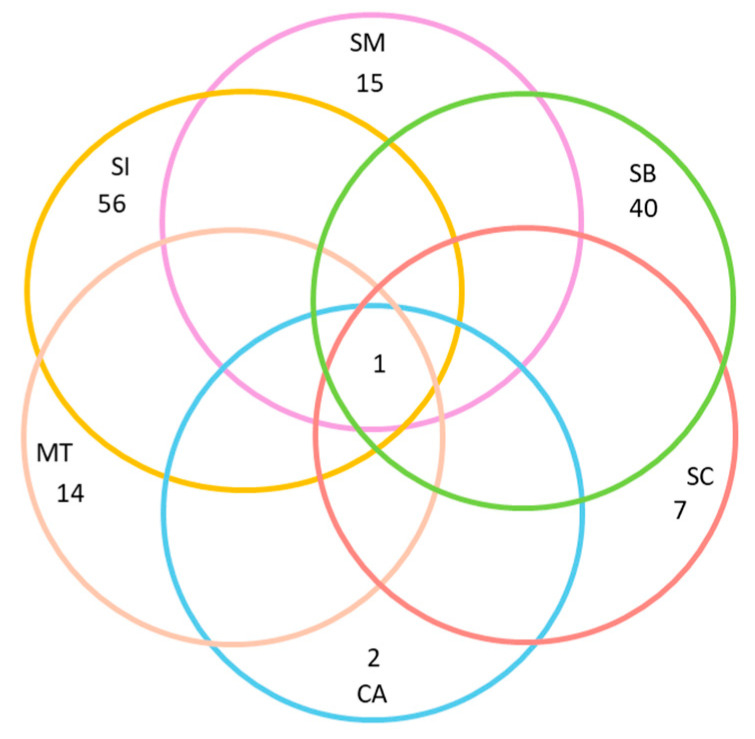
Venn Diagram representing unique lichen taxa growing on *Quercus ilex* in the six geographic areas considered. Colors represent different geographic areas: Montes de Toledo (MT), Sierra Morena (SM), Sistema Central (SC), Sistema Ibérico (SI), Sierras Béticas (SB), and Coastal Area (CA).

At both local and regional scales, the host preference accumulation curves initially displayed a steep slope during the early stages of sampling (Figure 2). As additional sites were incorporated (in our case, UTMs measuring 10 km × 10 km), the curve eventually stabilized, reaching an asymptotic state (Figure 2). Notably, this trend became even more pronounced when considering the entire dataset spanning all 486 UTMs. Thus, a larger surface area and a higher number of host species did not necessarily lead to a decrease in the prevalence of host preference. *Quercus suber*, *Q. ilex*, and *Juniperus thurifera* emerged as the host species with the highest number of lichen species restricted to a single host species. Lastly, the Venn diagram for *Quercus ilex* across the six geographic areas revealed a substantial number of exclusive species, particularly evident in Sierras Béticas and Sistema Ibérico (Figure 3). Examples of lichen species include *Catinaria atropurpurea* or *Crespoa crozalsiana* (Montes de Toledo), *Micarea misella* or *Ochrolechia dalmatica* (Sierra Morena), *Phaeophyscia endococcinea* or *Strangospora moriformis* (Sistema Central), *Gyalecta truncigena* or *Physma omphalarioides* (Sierras Béticas), *Scytinium fragrans* or *Usnea substerilis* (Sistema Ibérico), and *Alyxoria lichenoides* or *Parmelia hypoleucina* (Coastal Area). *Candelariella vitellina* was recorded on *Quercus ilex* in the six geographical areas considered.

## 4. Discussion

This is the first study to consider a large geographical area in the Mediterranean region to determine the true extent of host preferences, incorporating a high number of host species (72 species) and epiphytic lichens (709 species). This substantial number of lichen species represents a broad spectrum of the species in the peninsular Mediterranean region (over 70%), as there appears to be no bias due to a lack of studies on peninsular epiphytic lichens, which have received special attention for more than two centuries [24]. Furthermore, a high number of host species have been included in this study, providing a significant representation of Mediterranean vegetation. Additionally, to ensure comprehensive representation, all aspects have been considered holistically, without differentiating uses or stages of degradation of the forest or scrub, thereby providing a good representation of the Mediterranean landscape.

Our results indicate that the percentage of lichen species growing on a single host species is remarkably high, exceeding 30% locally (for each geographic area) and 25% regionally (across the entire data set). This is an interesting outcome for such an extensive study, especially considering that lichens are generally thought to utilize tree trunks only as a supporting substrate since they absorb water and nutrients from the atmosphere [36]. Indications of a certain degree of host preference have indeed been observed for all groups of organisms structurally dependent on the host (ref. in [37]). However, because epiphytic species primarily use the host as support, a weak host preference is assumed [37]. Moreover, several authors have pointed out that host biases are frequently inconsistent over large areas for cryptogamic epiphytes [38,39] because the effects of tree traits that potentially influence epiphyte performance are likely to be modulated by climate. For instance, while a low water-retention capacity in a given tree species’ bark may render it a poor host in a xeric habitat, the same tree species may be a good host in a mesic habitat [37]. However, when we consider the regional dataset, the host-preference accumulation curve demonstrates that a gradual increase in surface area does not imply a decrease in host-preference, but rather that it remains more or less constant. This phenomenon can be attributed to several causes: (1) hosts with very different types of bark (e.g., *Fagus*, *Juniperus*, *Pinus*, *Quercus*), as for instance, the physicochemical properties of *Quercus suber* are very different from the rest of the host species; (2) the wide climatic range of some species in the considered region (e.g., *Quercus ilex*); or (3) host species linked to extreme environmental conditions within the Mediterranean region (e.g., *Juniperus thurifera*).

The characteristics of bark, including texture, pH, and water-holding capacity, are often hypothesized to be crucial for the suitability of trees as hosts for cryptogamic epiphytes [4,7,10]. These factors may contribute to the presence of a large number of epiphytic exclusive species on *Quercus suber*, with 40 lichen species identified. The bark of the cork oak exhibits distinct anatomical and density characteristics compared to other host species, characterized by a rough and abrupt surface with numerous inlets and outlets that form deep cracks and cavities [40]. These distinctive bark characteristics result in a different floristic set (at least partially) compared to other host species. For example, several exclusive lichens growing on *Quercus suber* are crustose species with inconspicuous thalli (*Alyxoria culmigena*, *Bacidia propinqua*, *Eopyrenula leucoplaca*, *Micarea elachista*, *Mycoporum antecellens*, *Polyblastiopsis subericola*, *Porina coralloidea*, *Pseudosagedia borreri*) that colonize wide and deep cracks where moisture is retained longer. In fact, a study investigating the influence of bark roughness on epiphyte colonization demonstrated a correlation between bark roughness and lichen species composition for *Quercus suber* [40]. Furthermore, bark’s ability to absorb (‘water-holding’) and temporarily store (‘water-retention’) rainwater, determined by its porosity [41] and thickness [42], may enhance host quality. Several studies indicate that the water-holding retention capacity might play a role in host biases. For instance, the higher epiphytic loads of cyanolichens could be explained by the higher water-retention capacity of some host species over others. Chemical properties of the bark may also influence host preferences. For cryptogamic epiphytes, bark pH has been frequently correlated with host biases [5,38,43].

On the other hand, the composition of epiphytes also varies within the same host species across a wide climatic range. For instance, in *Quercus ilex*, which occurs in six geographic regions, the variation in epiphytes can be attributed to two interacting pathways: (1) interregional climatic variability and (2) bark water retention influenced by regional climate variations. Some researchers have observed that periods of rain or the duration of the dry season in the Mediterranean region lead to variations in bark thickness and density, affecting water retention (moistening) and consequently impacting epiphytic lichen composition [40]. This variability may partially explain the observed high diversity in lichen composition within the same host species across different geographic areas. For example, species with higher water requirements, such as *Leptogium cyanescens*, *L. magnussonii*, *Nephroma parile*, *Sticta fuliginosa*, or *Usnea substerilis*, which grow on the bark of *Quercus ilex*, were more frequently recorded in the northern regions of the Sistema Ibérico, where precipitation levels are higher. However, species of the genera *Phaeophyscia*, *Physcia*, and *Physconia* were more abundant in the drier regions of the southeastern Sistema Ibérico and Sierras Béticas.

Finally, a special case related to *Juniperus thurifera*, one of the most distinctive formations in Western Europe, can be considered. This species thrives under continental climatic conditions, characterized by significant temperature contrasts between day and night, as well as between winter and summer. Its optimal climatic conditions are found in the northeastern quadrant of the Peninsula [24]. *Juniperus thurifera* tends to develop on open structures and in monospecific woodlands, often confined to hard limestone areas with limited water availability [24]. These challenging environmental conditions, including wide thermal ranges and colder temperatures, promote lichen specialization. Consequently, specific groups of crustose lichen species have adapted by minimizing their thallus size in these harsh environments. These inconspicuous crustose species are well-suited to colonize the hard and acidic bark of juniper trees [44]. Thus, species such as *Biatora ocelliformis*, *Lecanora paramerae*, *Pertusaria parameae*, and *Chaenotheca trichialis* were more frequently found in the continental juniper forests.

In this study, we exclusively analyzed the host preferences of epiphytic lichens across diverse phorophyte host species without considering other factors. On a broader scale, climate may significantly influence these preferences, especially where only a single host tree species is present. For example, more stressful, drier climatic conditions pose a significant threat to epiphytic lichen communities due to the loss of generalist species [45]. Under these conditions, desiccation-tolerant species are favored, leading to the presence of specific lichen groups, such as crustose species with inconspicuous thalli. Thus, some species of the genera *Arthonia*, *Lecanora*, or *Rinodina* inhabit a low number of host species. Conversely, more favorable climatic conditions promote the dominance of generalist species [27,39,45]: in our study, parmelioid lichen species. This study serves as a foundation for further exploration of the various aspects related to lichen diversity patterns. Addressing these issues is essential for enhancing our understanding of their potential applications in the conservation of these organisms.

## 5. Conclusions

This study reveals a remarkable consistency in host bias among epiphytic lichen species across Mediterranean landscapes. Despite expanding the sampling area, the proportion of lichens species associated with a single host species remained constant at 25%. This persistence underscores the intricate and resilient relationships between lichens and their host species. The Mediterranean landscape, with its rich diversity of tree and shrub species, historical land use patterns, varying conservation states, and significant climatic range within a narrow geographical area, fosters a diverse array of epiphytic species. These factors are pivotal in driving the high levels of host preference observed in lichen species, highlighting the complexity and specificity of these ecological interactions. Our findings emphasize the importance of considering host preferences in conservation strategies to preserve the unique biodiversity of Mediterranean epiphytic lichens.

## Figures and Tables

**Table 1:** General data on the number of UTMs, host species, and lichen species across the six geographic areas considered. ps: phorophyte species; tls: total lichen species; rsps: lichen species restricted to a single phorophyte species.

Geographic Area	No. UTM	No. ps	No. tls	rsps (%)
Local scale (geographic area)				
Montes de Toledo	44	13	283	36.40
Sierra Morena	45	19	296	35.14
Sistema Central	92	29	327	32.11
Sierras Béticas	75	36	456	37.28
Sistema Ibérico	188	38	473	32.14
Coastal Area	38	14	145	50.34
Regional scale				
Total	486	72	709	25.90

**Table 2:** Host species with at least five excusive lichens species at the regional scale (i.e., considering the entire dataset). tls: total lichen species; rsps: lichen species restricted to a single phorophyte species.

Regional	No. UTM	No. tls	No. rsps	rsps (%)
*Quercus suber*	50	337	40	8.9
*Quercus ilex*	202	342	21	6.1
*Juniperus thurifera*	48	154	13	7.4
*Fagus sylvatica*	19	193	11	5.7
*Juniperus oxycedrus*	30	152	10	6.5
*Quercus pyreniaca*	109	295	9	3.1
*Pinus pinaster*	39	169	8	4.7
*Olea europea*	28	151	8	5.3
*Erica australis*	6	88	7	7.9
*Quercus faginea*	109	250	7	2.8
*Abies pinsapo*	6	119	5	4.2

## Data Availability

The dataset is available on ZENODO (https://doi.org/10.5281/zenodo.14753452).

## Supplementary Materials

The following supporting information can be downloaded at: https://www.mdpi.com/article/10.3390/jof11020104/s1. Table S1: Epiphytic lichen species and phorophyte species across the studied area.
